# Larvicidal effect of disinfectant soap on *Anopheles gambiae* s.s (Diptera: Culicidae) in laboratory and semifield environs

**DOI:** 10.1186/1756-3305-7-211

**Published:** 2014-05-03

**Authors:** France P Mdoe, Gamba Nkwengulila, Mariam Chobu, Lucile Lyaruu, Israel L Gyunda, Saada Mbepera, Rui-De Xue, Eliningaya J Kweka

**Affiliations:** 1 Department of Zoology and Wildlife Conservation, College of Natural and Applied Sciences, University of Dar-es-salaam, P.O.Box 35165 Dar-es-salaam, Tanzania; 2 Division of Livestock and Human Diseases Vector Control, Tropical Pesticides Research Institute, Ngaramtoni, Off Nairobi Road, P.O. Box 3024 Arusha, Tanzania; 3 Anastasia Mosquito Control District, 500 Old Beach Road, St. Augustine, FL 32080, USA; 4 Department of Medical Parasitology and Entomology, Catholic University of Health and Allied Sciences, P.O. Box 1464, Mwanza, Tanzania

**Keywords:** *Anopheles gambiae* s.s, Larvicidal, Semi field, Laboratory, Microcosms

## Abstract

**Background:**

Mosquito larval control using chemicals and biological agents is of paramount importance in vector population and disease incidence reduction. A commercial synthetic disinfectant soap was evaluated against larvae of *Anopheles gambiae* s.s. in both laboratory and semi field conditions.

**Method:**

Five concentrations of commercial synthetic disinfectant soap (0.0001, 0.001, 0.01, 0.1 and 1%) were prepared and evaluated against third instar larvae in laboratory and semi field environments. Mortality was scored at 12, 24, 48, and 72 hrs. Each dosage had 6 replicates, having twenty 3^rd^ instar larvae of *An.gambiae* s.s.

**Results:**

In the laboratory phase, all dosages had significantly higher larval mortalities than in controls, while in semi field conditions, the dosages of 0.0001, 0.001 and 0.01% had lower mortalities than laboratory trials. In the comparison between semi field and laboratory trials, only 0.1 and 1% dosage had significant difference with more mortality in semifield conditions. Proportions of larvae that died during mortality monitoring intervals in laboratory and semi field had significant differences only at 12 hrs and 72 hrs.

**Conclusion:**

The findings of this study have demonstrated that the mortality of larvae caused by commercial synthetic disinfectant soap is worth further studies in open water bodies. More studies are necessary to find out the effect of sunlight on the chemistry of the synthetic disinfectant and other variables in small scale full field trials.

## Background

The anthropophilic malaria vector, *Anopheles gambiae* s.s is the most efficient vector in sub-Saharan Africa [1]. Control measures such as long lasting insecticide treated bed net (LLINs) and indoor residual spray (IRS) have been successful in reducing malaria disease burden [2,3]. Currently the spread and rise of insecticide resistance have jeopardized the efficacy of these tools [4-8]. Increasing vector control efforts targeting larval sources is of high priority as larvae are relatively immobile compared to adult mosquitoes [9-12]. Controlling mosquitoes using larviciding has shown a great impact in larval mortality in field situations [13]. Environmentally friendly compounds with high larval mortality effect are currently needed. Due to environmental concerns about the safety of pesticides, the interest of revisiting the use of insecticidal soap has been raised. Fleas, ticks and cockroaches are among arthropods significantly controlled by the use of disinfectant soap [14]. Some of these soaps formulations have proved to be effective on cockroach mortality [15]. Abbasi and others demonstrated the efficiency of commercially available disinfectant soaps against crickets and cockroaches [14]. The use of soap in insect control is among the “old days methods” [16], though the utilization of antibacterial soaps have not been screened against insect pests, mostly *An. gambiae* s.s. Further efforts are needed for the investigation of soaps and detergents for control of mosquito larvae of different species in human dwellings structures including in swimming pools and septic tanks.

The objective of this study was to determine mosquito larvicidal activity of commercially available synthetic disinfectant soap against *An. gambiae* s.s in both laboratory and semi field environments.

## Methods

### Mosquito colony

Mosquitoes originated from a colony of *An. gambiae* s.s established from Kisumu Kenya in 1992 and were reared at the tropical pesticides Research Institute (TPRI). Laboratory rearing of larvae was as described in other protocols [17,18]. In the insectary larvae were fed with tetramin fish food at rate of 0.003gm/larvae. Third instar larvae were used for trials as recommended in WHO protocol [19]. The photo phase in the insectary was 12Light: 12Darkness (12 L: 12D) with a temperature of 27 ± 2 °C and Relative humidity of 78 ± 2%.

### Larval bioassays in insectary

Five concentrations of commercial synthetic disinfectant soap were prepared; 1, 0.1, 0.01, 0.001, and 0.0001%. The stock solution was made using distilled water. Experimental solutions were obtained through serial dilutions. Serial dilutions were made as described in the WHO larval bioassay protocol [19]. Small bowls with diameter of 14 cm, depth 10 cm, and a capacity of 250mls were used as microcosms during experiments. Each replicate had a total of twenty larvae. All five concentrations above had an effect on mortality and were considered for laboratory bioassays. Mortality data were recorded at 12, 24, 48 and 72 hours after experimental set up for both control and treatments. The moribund larvae were considered dead.

### Semi field larval bioassays

**Figure 1:**
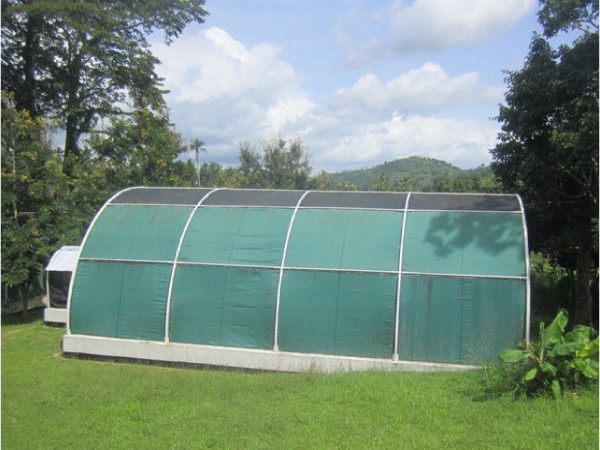
A picture of Semifield structure.

The semi field structure environment has been constructed to mimic local, outdoor conditions [20]. The semi field structure has a dimension of 12.2 m long and 8.2 m wide (Figure 1), allowing wind flow, precipitation and creating similar climatic conditions to ambient conditions. Entrance into the sphere is through a double door system; a wooden door provides entrance to the sphere, after passing through a small corridor (3.0mlong and 2.2 m wide) covered with plastic transparent roofing material, with a screened door to the outside. This prevents escape of released mosquitoes and entry of wild mosquitoes. There is some vegetation grown in semifield structures to mimic the natural land cover. Semi field protocol was used as described in WHO manual [19]. The same dosages used in the laboratory were used in semi field. The main difference with laboratory trials, in semifield the microcosm was exposed to sunlight and night weather without control of any parameter such as temperature and light. Semi natural environment conditions are paramount in understanding the efficacy of the evaluated compounds in complex environments as compared to reported efficacy in the laboratory against larvae of *An.gambiae* s.s.

### Disinfectant soap

The disinfectant soap used was liquid soap often used for domestic cleaning. It includes triclosan (Irgasan), 97% granular, as the active ingredient purchased from Sigma- Aldrich (St. Louis, MO, AC abstract 3380-34-5). It was once evaluated against larvae of *Cx. quinquefasciatus* mosquitoes and proved to have a lethal effect [21].

### Data analysis

Percentage mortality was corrected by abbot’s formula [22]. Analysis of variables (ANOVA) was used to calculate the mean percentage mortality and standard error in different concentration and hours. Chi-square test was used to calculate the statistical significant difference between the proportions of larvae that died in control and treatment groups and also the proportion that died in the hours between control and treatment. Data analyses were performed with Statistical programme for social scientist (SPSS) version 18.0 for windows (SPSS Inc, Chicago, IL).

## Results

### Laboratory bioassays

In laboratory evaluations, all dosages had higher mortality in treatments than control (Table 1). The proportion of mosquito larvae that died in each time interval was significantly higher in treatments than in control (Table 2).

**Table 1:** **Larvicidal effect of different disinfectant soap dosages on *Anopheles gambiae *s.s in laboratory and semi field conditions for treatment and control**

**Experiments**	**Dosage (%)**	**Treatment**	**Control**	**Χ**^**2 **^**(P-value)**
		**% Mortality (Mean ± SE)**	**% Mortality (Mean ± SE)**	
Laboratory	0.0001	3.1 ± 3.1	3.8 ± 2.4	173.36 (<0.001)
	0.001	10.6 ± 7.9	2.5 ± 2.5	5.36 (0.021)
	0.01	13.8 ± 9.4	5.0 ± 2.9	4.55 (0.033)
	0.1	57.7 ± 2.4	2.5 ± 1.4	72.41 (<0.001)
	1	93.3 ± 6.7	7.5 ± 2.5	147.42 (<0.001)
Semi-field	0.0001	0.00 ± 0.00	6.3 ± 1.3	NC
	0.001	0.00 ± 0.00	0.00 ± 0.00	NC
	0.01	0.6 ± 0.4	2.5 ± 1.4	NC
	0.1	83.3 ± 11.8	7.5 ± 2.5	115.89 (<0.001)
	1	97.3 ± 2.7	11.3 ± 3.8	136.14 (<0.001)

Note: NC-Not calculable; Each mean is the mean of six replicates of 20 larvae each.

**Table 2:** **Larval mortality in treatment (water treated with disinfectant soap) and control on *Anopheles gambiae *s.s in laboratory and semi field conditions within different monitoring hours**

**Experiments**	**Time**	**Treatment**	**Control**	**Χ**^**2 **^**( *P *-value)**
	**(in hours)**	**% Mortality (Mean ± SE)**	**% Mortality (Mean ± SE)**	
Laboratory	12	14.67 ± 14.67	0.00 ± 0.00	15.83 (<0.001)
	24	27.83 ± 19.60	2.00 ± 2.00	26.29 (<0.001)
	48	43.17 ± 21.67	6.00 ± 1.87	37.26 (<0.001)
	72	57.17 ± 18.07	9.00 ± 1.00	52.40 (<0.001)
Semi-field	12	27.8 ± 18.1	2.0 ± 1.2	26.25 (<0.001)
	24	36.7 ± 22.6	7.0 ± 2.5	25.83 (<0.001)
	48	40.2 ± 24.4	8.0 ± 2.0	28.34 (<0.001)
	72	40.3 ± 24.4	9.0 ± 1.9	26.37 (<0.001)

Note: Each mean is the mean of six replicates of 20 larvae each.

### Semi field bioassays

In semi field bioassays there was no mortality in low dosages of 0.0001 and 0.001, while in 0.01% mortality was 0.6% which was low and had no results when a chi-square test was used to compare treatment and control results. The mean mortalities were statistically higher in treatment than in control (Table 1). The proportion of larvae that died in each time interval was significantly higher in treatment than in control (Table 2).

### Comparison of laboratory and semi field bioassays of disinfectant soap

**Figure 2:**
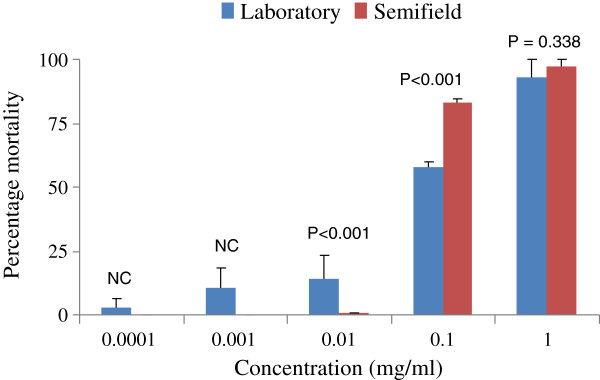
**Percentage mortality of *An. gambiae *** s.s larvae in different concentrations of disinfectant soap in between laboratory and semi field experiments (**NC-Not calculable).

**Figure 3:**
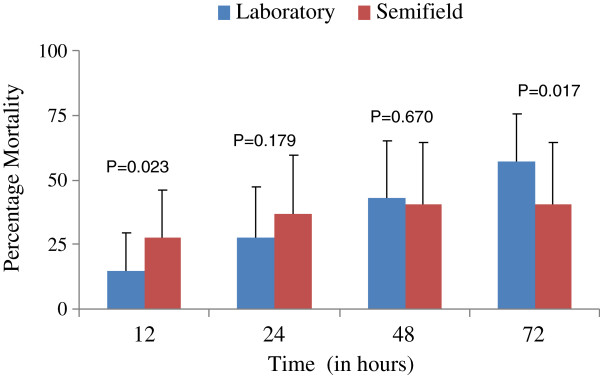
**Mortality response percentage of *An. gambiae *** **s.s larvae in monitoring time intervals in between laboratory and semi field experiments.**

The comparison of mean mortality in all dosages varied. In the lowest dosage of 0.0001 and 0.001% there was no larval mortality in semi field conditions, hence no mortality variations among replicates. The rest of the dosages had significantly higher differences which varied, at dosage of 0.01% more larvae died in the laboratory than in semi field. In the rest of the higher dosages, more larvae died in semi field than in laboratory conditions (Figure 2). Mortality was found to be significantly different between laboratory and semi field only at 12 and 72 hrs for all dosages (Figure 3).

## Discussion

The findings of this study have shown that at low dosages of 0.1% and 1% mortalities of 93.3 and 97.3% in the laboratory and semi field respectively were observed. Most of these disinfectant soaps contain alkyls, chlorides and alcohols which do not show significant larvicidal activity against mosquito larvae but only when application is made in higher dosages [21].

Our results have shown that, larval mortality was dosage dependant for disinfectant soap against *An. gambiae* s.s, which was similar to what was found in *Cx. quinquefasciatus* by Subra and others [23]. In the laboratory all dosages had significant larvicidal impact relative to control. The same dosages in a semi field environment had induced mortality only in the two highest dosages of 0.1 and 1%. The low dosages of 0.0001 up to 0.01% had no appreciable larvicidal effect in a semi field environment. This might have been attributed by the exposure of disinfectant soap under sunlight which might have broken it down into secondary metabolite products which had low toxicant effect, but higher dosages such as 1% could still show higher mortality. A similar scenario was observed when low dosages of *Schinus terebinthifolia* (radii) could not show larvicidal effect on *An. gambiae* s.s in semi field conditions [24]. However, in monitoring mortality, the semi field results showed higher mortality at an earlier monitoring time of 12 and 24 hrs while in the insectary it was in 48 and 72 hrs. The semi field results might be attributed to degradation of the chemical structure of compounds hence causing no larvicidal effect with further degradations of compounds into secondary metabolites. In the laboratory the rate of degradation is low for active ingredients hence higher mortality effects are noticed throughout monitoring time. This trend was similar to the findings in other screened larvicides [11,24]. *An. gambiae* are surface biofilm feeders, which might have been attributed to the observed reduced mortality while *Ae. Aegypti* and *Cx. quinquefasciastus* are bottom feeders, which causes them to feed on high amounts of synthetic disinfectant [25]. Based on this feeding behaviour, the previous findings had higher mortality in bottom feeders than observed *An.gambiae* s.s mortalities. This might be attributed with the sedimentation of the particles at the bottom that could increase mortality in bottom feeding species than it could for surface biofilm feeders where concentrations of the disinfectant soap is decreasing with time.

The main active ingredient of the disinfectant soap evaluated for larvicidal activity on *An. gambiae* s.s was triclosan. This product has shown a significant insecticidal activity against cockroaches [14,15] and scape insects [16] and ticks [26]. Currently, there are two reported findings which have shown insecticidal activity of soap products against mosquitoes [23].

The results of the current study on disinfectant soap containing triclosan had 3.1 to 93.3% mortality in the laboratory and 0.0 to 97.3% in semi field evaluation. These results are by margin lower than previous trials with *Cx. quinquefasciastus* using the same disinfectant. Understanding of disinfectants for effective larvicidal outcome on *An. gambiae* s.s is of priority for larval control.

The domestic disinfectant soaps have proved to have low eco-toxicological impact and have been shown to be safe for domestic insect control [14]. The study conducted by Xue and Qualls has shown that, the domestic soap with triclosan had increased mortality effects on mosquitoes compared to previous studies which did not include triclosan as active ingredient [21]. The most important part of the research is to understand the effect of triclosan to non-targeted organisms in larval habitats.

The need of new and innovative methods for mosquito control is currently increasing due to high insecticide resistance in disease vector mosquitoes [4-8] and behavoural changes due to control tool implementation [27]. To increase the process of shrinking malaria and vector populations, the targeted sources reduction is of priority. Development of larvicides and screening of their bioefficacy is important for effective control. Screening for better larvicides is ongoing in different parts of malaria and non-malaria endemic regions [11,24].

## Conclusion

The findings of this study have demonstrated that the mortality of larvae shown by disinfectant soap is worth for further studies in open water bodies. More studies have to find out the effect of sunlight and other variables in semi field before small scale field trials. Other mosquito species should be included in further trials.

## Competing interests

Authors declare to have no competing interest.

## Authors’ contributions

EJK and RX conceived the study. EJK and FPM designed experiments. FPM, MC, LL, IG, SM performed experiments. FPM did data analysis and interpretation. EJK, RX, GN and FPM wrote the manuscript. All authors reviewed the draft and accepted it for submission. All authors read and approved the final manuscript.
